# Up-regulation of CXCL8 expression is associated with a poor prognosis and enhances tumor cell malignant behaviors in liver cancer

**DOI:** 10.1042/BSR20201169

**Published:** 2020-08-20

**Authors:** Song Yang, Han Wang, Chundi Qin, Hongmei Sun, Yupeng Han

**Affiliations:** 1 First Department of Pediatrics, First Affiliated Hospital of Jiamusi University, Jiamusi 154002, Heilongjiang, P.R China; 2 Department of Gastroenterology, Affiliated Hospital of Inner Mongolia Medical University, Hohhot 010050, Inner Mongolia, P.R China; 3 Second Hospital of Heilongjiang, harbin 150000, Heilongjiang, P.R China; 4 First Department of Oncology, Oncology Hospital of Jiamusi, Jiamusi, Heilongjiang 154002, P.R. China; 5 First Department of Gastroenterology, First Affiliated Hospital of Jiamusi University, Jiamusi 154002, Heilongjiang, P.R China

**Keywords:** CXCL8, ERK, Inflammation, liver cancer, malignant progression

## Abstract

CXCL8, a member of CXC chemokines, was constitutively expressed in many types of human cancers, and its overexpression has been shown to play a critical role in promoting tumorigenesis. The purpose of the present study was to determine CXCL8 expression in a commercial human liver tissue microarray, and elucidate the effects and underlying mechanisms by which CXCL8 is involved in the malignant progression of human liver cancer. Our data showed that high level expression of CXCL8 in tissues with liver cancer was identified as compared with non-cancer tissues, and its up-regulation was closely associated with clinical stage and tumor infiltration. *In vitro*, exogenous CXCL8 at concentrations of 10, 20 or 40 ng/ml obviously stimulated the proliferation abilities of HepG2 cells. Coupled with this, 10, 20 or 40 ng/ml of exogenous CXCL8 also triggered a significant elevation in HepG2 cells migration. Additionally, overexpression of CXCL8 in HepG2 cells also resulted in increased cell proliferation and migration capacities. Finally, Western blotting analysis showed that overexpression of CXCL8 increased the expression of ERK, p-ERK and survivin, decreased the expression of caspase-3 and BAX at protein level.

## Introduction

Liver cancer, the predominant primary malignancy, ranks as the fifth most commonly diagnosed cancer in male and the seventh in female around the world [1]. In the year 2018, it has been estimated that liver cancer is the fourth leading cause of cancer death, with approximated 841,000 new cases were diagnosed, and over 782,000 patients succumbed to this malignancy [2]. The incidence of liver cancer differs among different geographical regions, and the global map of liver cancer revealed that it remains a highest incidence rates in East Asia, with China accounting for more than 50% of the burden in the world [2,3]. Multiple risk factors, including chronic infection with hepatitis B virus (HBV) or hepatitis C virus (HCV), aflatoxin-contaminated eatables intake, heavy alcohol consumption, smoking, corpulence, and Type 2 diabetes, are suggested to be essential for the occurrence of liver cancer [4,5]. Of the two infection diseases, HBV is suggested to be implicated in 75–80% of virus-associated liver cancer, while HCV is implicated in 10–20%, suggesting a predominant role of inflammation/immune response in the process of liver cancer [6].

Chemokines, also named as chemoattractant cytokines, are a large subfamily of small heparin-binding proteins and originally characterized by their properties to direct and recruit the movement of various leukocyte subsets [7]. On the basis of the position of the cysteine residues adjacent to the N-terminal, chemokines were classified into four conserved subfamilies (namely CC, CXC, CX3C and C), and they execute their contributions mainly through interactions with their G-protein-coupled receptors, identified as CCR, CXCR, CX3CR and CR [8]. It is generally acknowledged that chemokines and their receptors may play multifaceted roles in engendering and mediating inflammation and immune response, and therefore be involving in the pathogenesis and pathological process of various diseases [9]. Recently, expanding evidence highlighted that chemokines/receptors were constitutively expressed and responsible for various malignant progression, including tumor cell proliferation, migration, invasion, metastasis and tumor angiogenesis, in a variety of human tumor types [10].

In the current research, we evaluated whether CXCL8, a member of CXC chemokines, was constitutively expressed in the tissues with liver cancer, and explored the underlying role and mechanism of CXCL8 in regulating malignant progression of liver cancer.

## Materials and methods

### Cel line, cell culture and cell transfection

The human hepatoblastoma cell line HepG2 were provided by American type culture collection (ATCC, U.S.A.), and the cells were cultured in RPMI-1640 medium supplemented with 10% fetal bovine serum (FBS; Gibco; Thermo Fisher Scientific, Inc.) containing 100 units/ml penicillin and 100 mg/ml streptomycin at 37°C with 5% CO_2_ in a humidified atmosphere. A expression plasmid carrying CXCL8 were applied to transfect cells using Lipofectamine 2000 (Invitrogen) according to the supplier’s instructions, and the transfection efficiency was verified using a human CXCL8 ELISA kit (Boster Biological Technology, Wuhan, China) following the manufacturer’s recommendations.

### Immunohistochemistry staining assay

A commercial human tissue microarray containing 40 liver cancer and 40 normal liver tissue samples (Alenabio, Xi'an, China) was used to estimate the expression of CXCL8. Sample characteristics were shown in Table 1. After routine deparaffinization and hydration, the microarray was treated with 0.3% (v/v) hydrogen peroxide/methanol for 20 min at room temperature for inactivating endogenous peroxidase activity. Following antigen retrieval with 0.01 M citrate buffer in a microwave oven for 18 min, the microarray was blocked with normal goat serum for 45 min at room temperature and subsequently probed with a primary antibody specific for CXCL8 (dilution, 1:50). After rinsing with PBS, the microarray was incubated with HRP-conjugated secondary antibody (Boster Biological Technology, Wuhan, Hubei, China) at 37°C for 30 min. Finally, DAB substrate was utilized for the visualization of antigen, and haematoxylin for a routine nuclear counterstain. The expression of CXCL8 at protein level was evaluated as the following two parameters. (a) The expression intensity: 0 = negative; 1 = weak; 2 = mild; 3 = strong, 4 = super strong staining. (b) The percentage of cells stained: 1 = <25%; 2 = 25–50%; 3 = 50–75%; 4 = >75% of staining. The scores for the two parameters are summed to produce a total score. A total score of ≤ 4 was referred to as low expression, whereas ≥ 5 as high expression.

**Table 1:** Sample characteristics in a liver tissue microarray

Characteristic	Cancer samples (case)	Normal samples (case)
Gender		
Male	34	34
Female	6	6
Age, year		
Range	30-70	35-70
Median	50.85	46.50
Stage		
II	16	
III	22	
IV	2	
Grade		
1	6	
2	14	
3	20	

### Cell proliferation assay

HepG2 cells at density of 2 × 10^3^ per well were maintained in 96-well plates in 100 μl of RPMI 1640 medium containing 10% FBS with or without exogenous CXCL8. Following culture for up to 24, 48 or 72 h, a cell proliferation assay kit (counting kit-8, Boster Biological Technology, Wuhan, China) was applied to determine the proliferation ability of cells following the manufacturer’s recommendations. The optical density (OD) in individual wells was detected with a microplate reader at 450 nm.

### Cell migration assay

A total of 2 × 10^4^ HepG2 cells were individually maintained in each chamber of 8-μm pore inserts containing 100 μl of serum-free RPMI 1640 medium, and the low chamber, which contained 600 μl of RPMI 1640 medium supplemented with 10% FBS with or without exogenous CXCL8 was employed as attractant. After 24 h incubation, the cells that had penetrated through the inserts and attached to lower surface of the inserts were fixed and stained using 95% ethanol containing 1% viola crystallina. Finally, the migrating cells were photographed and counted under a light microscope.

### Apoptosis assay

Cell apoptosis was detected using a commercial an apoptotic-Hoechst staining kit (Beyotime, Shanghai, China). Briefly, the cells were fixed with fixative solution, and the cells were stained with Hoechst 33258 followed by washing with PBS three times. A confocal microscope was used to determined cell apoptosis, the apoptotic rate was evaluated by the following formula:

$$\text{Apoptotic rate }(\%)=\frac{\text{total number of cells }-\text{ number of apoptotic cells}}{\text{total number of cells}}\times 100\%$$

### Western blotting assay

Total protein from individual cell pellets was extracted using a commercial RIPA lysate buffer (Boster Biological Technology, Wuhan, China) following supplier’s instructions. About 30 μg of total protein was subjected to electrophoresis in 12% SDS-PAGE and subsequently electrotransferred onto a PVDF membrane (Millipore, Bedford, MA). After blocking with 5% non-fat milk at room temperature for 1 h, the membrane was, respectively, incubated overnight at 4°C with different specific antibodies directed against ERK (1:1000, Abways), p-ERK(1:1000, Abways), survivin (1:500, santa cruz), BAX (1:500, santa cruz), caspase-3 (1:1000, affinity) and β-actin. HRP-linked secondary antibodies and ECL reagent were used to display protein bands. The relative protein levels were normalized against β-actin.

### Statistical analysis

Data were analyzed using SPSS 20.0 software. *In vivo*, the Pearson Correlation Coefficient analysis was applied to reveal significant differences among several clinicopathological parameters. *In vitro*, data were presented as means ± standard deviations, significant differences between two groups were compared using the Student’s *t*-test. *P*<0.05 was considered as statistical significance.

## Results

### CXCL8 was overexpressed in tissues with liver cancer

**Figure 1:**
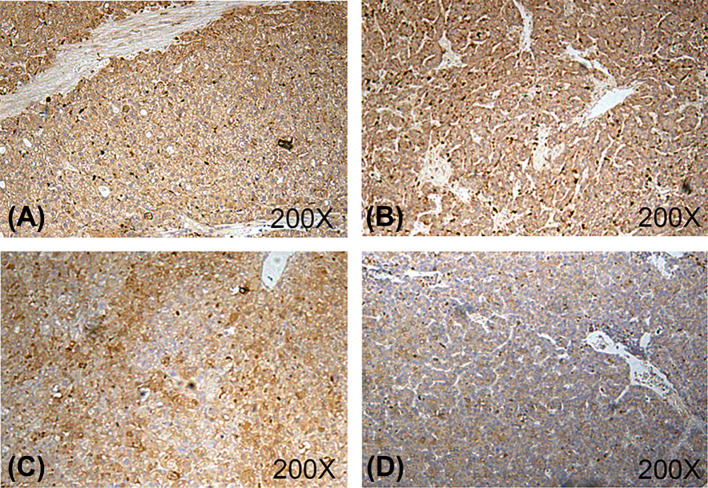
Up-regulated expression of CXCL8 in tissues with liver cancer in a human liver tissue microarray Liver cancer tissues with stage II (**A**), III (**B**) and IV (**C**) represented strong staining of CXCL3. Normal tissue (**D**) showed weak staining of CXCL8.

The results from immunohistochemistry assay showed that the expression of CXCL8 at protein level was markedly increased in liver cancer tissues (Figure 1A–C), whereas normal liver tissue showed a decreased expression of CXCL8 protein (Figure 1D) (*P*=0.0246). In addition, statistical analysis form liver cancer tissues demonstrated that up-regulated level of CXCL8 was positively concerned with high clinical stage and tumor infiltration (*P*=0.0061). On the contrary, there was no positively association between CXCL8 expression and other clinicopathological parameters including pathological grade, sex and age (Table 2).

**Table 2:** CXCL8 level in a liver tissue microarray

Characteristic		Case of score≤ 4 (%)	Case of score≥ 5(%)	χ^2^	*P*
normal liver samples		27(67.5%)	13(32.5%)	5.0505	0.0246
HCC samples		17(42.5%)	23(57.5%)		
Clinical stage/Tumor	II /T2	11(68.75%)	5(31.25%)	7.5192	0.0061
infiltration(T)	III-IV/T3-T4	6(25.0%)	18(75.0%)		
Pathological grade	1-2	10(50.0%)	10(50.0%)	0.9207	0.3373
	3	7(35.0%)	13(65.0%)		
Age	≤ 50	10(45.5%)	12(54.5%)	0.1746	0.6760
	>50	7(38.9%)	11(61.1%)		
Sex	Man	15(44.1%)	19(55.9%)	0.2427	0.6223
	Woman	2(33.3%)	4(66.7%)		

### Exogenous CXCL8 regulates proliferation, migration and apoptosis of HepG2 cells

**Figure 2:**
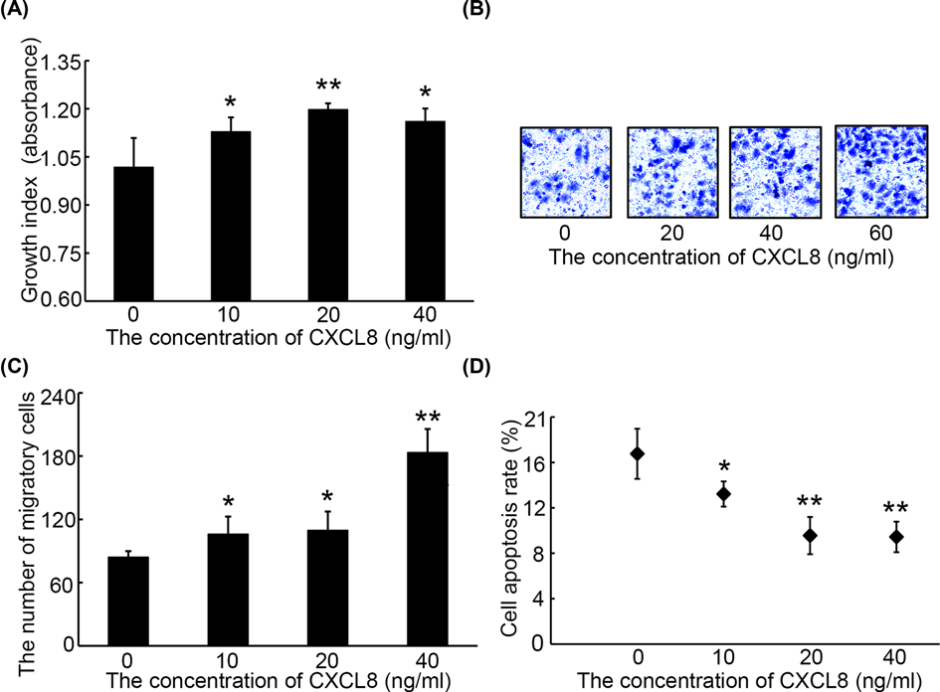
The effects of exogenous CXCL8 in cell proliferation, migration and apoptosis of HepG2 cells *in vitro* Proliferation index (**A**), the number of migration (**B** and **C**) and apoptosis rate (**D**) from HepG2 cells following treatment with exogenous CXCL3 at different concentrations. *P<0.05, **P<0.01 versus 0 ng/ml of CXCL8.

To assess the roles of CXCL8 in regulating malignant behavior of liver cancer, a classic cell Line namely HepG2 was employed for following cell experiments. CCK-8 analyses demonstrated that the growth rate of HepG2 cells treated with 10, 20 or 40 ng/ml CXCL8 was significantly elevated as compared with HepG2 cells treated with 0 ng/ml CXCL8 (Figure 2A). Coupled with this, transwell analyses revealed that migration cells of HepG2 cells treated with 10, 20 or 40 ng/ml CXCL8 was significantly more than that of HepG2 cells treated with 0 ng/ml CXCL8 (Figure 2B,C). In addition, apoptosis assay showed that treatment of cells with 10, 20 or 40 ng/ml CXCL8 can significantly inhibit the apoptosis rate of cells (Figure 2D).

### Overexpression of CXCL8 contributes to proliferation, migration and apoptosis of HepG2 cells

**Figure 3:**
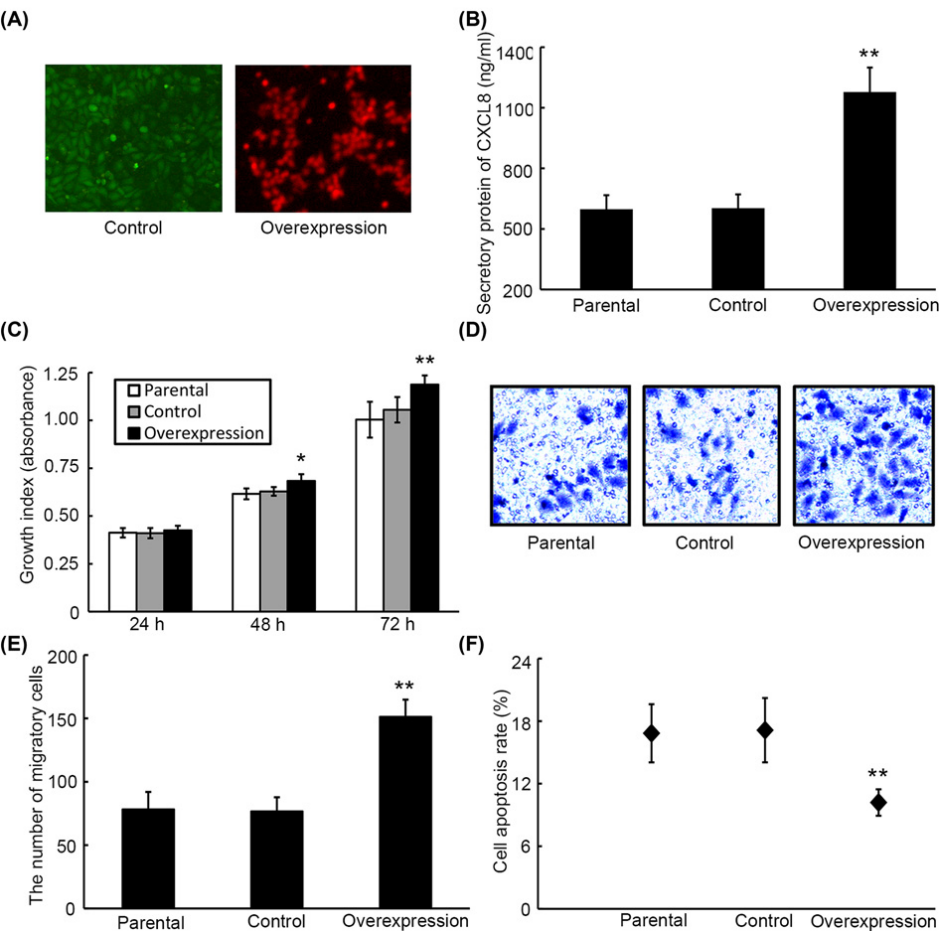
The effects of overexpression of CXCL8 in cell proliferation, migration and apoptosis of HepG2 cells *in vitro* (**A**) Fluorescence images of CXCL8-overexpression cells and their control cells. (**B**) The expression of CXCL 8 in supernatant from CXCL8-overexpression cells, parental cells and control cells was detected by ELISA. The proliferation index (**C**), number of migration (**D** and **E**) and apoptosis rate (**F**) from HepG2 cells overexpresssing CXCL8, parental cells and control cells. *P<0.05, **P<0.01 versus control.

To reveal the role of CXCL8 in liver cancer, gene transfection strategy was used to construct CXCL8-overexpressing HepG2 cells and their control. Fluorescent detection demonstrated that cells exhibited high content of fluorescence (Figure 3A). Moreover, ELISA analysis showed that the level of CXCL8 in supernatant from CXCL8-overexpression cell medium was remarkably increased as compared with supernatant from their parental and control cell medium, indicating HepG2 cells overexpressing CXCL8 have be established (Figure 3B). CCK-8 and transwell assays demonstrated that the proliferation (Figure 3C) and migration (Figure 3D,E) abilities of HepG2 cells overexpressing CXCL8 were markedly enhanced as compared with their parental and control cells. On the contrary, the apoptosis rate of overexpression cells was significantly lower than those of parental and control cells (Figure 3F).

### Overexpression of CXCL8 regulates tumor-specific protein expression of HepG2 cells

**Figure 4:**
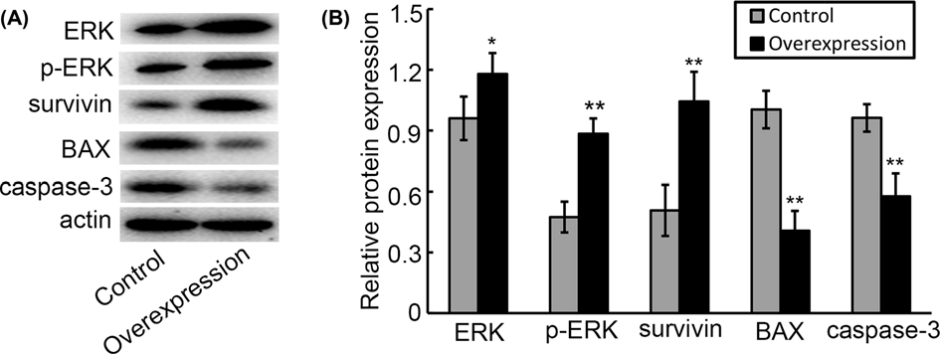
The effects of CXCL8 in regulating tumor-specific protein expression (**A**) Representative images of western blotting analysis for ERK, p-ERK, survivin, caspase-3 and BAX genes. (**B**) Relative expression of these genes at protein levels from Western blotting analysis. *P<0.05, **P<0.01 versus control.

Finally, we investigated whether overexpression of CXCL8 regulates the expression of tumor-related genes, the data from Western blotting indicated that CXCL8-overexpression cells presented significantly increased levels of ERK, p-ERK and survivin compared with their control cells. Inversely, the levels of caspase-3 and BAX in CXCL8-overexpression cells were predominantly reduced than that in their control cells (Figure 4A,B).

## Discussion

CXCL8, also known as IL-8, is a member of the C-X-C family of chemokines that is produced by many types of cells including leukocytes, fibroblasts, endothelial cells and malignant cancer cells, and play a various spectrum of biological effects in cell functions through interaction with its G protein-coupled receptors, CXCR1 and CXCR2 [11,12]. CXCL8 are best known for their function in the initiation of the inflammatory reaction [13]. During the process of inflammation, CXCL8 recruits leukocytes to the site of infection, ultimately resulting in increased neutrophil infiltration, which is responsible for the damage of endothelial cells. This implies that down-regulation of CXCL8 is crucial for the resistance to chronic inflammation [14].

In addition to its function in inflammation and immune response, increasing evidences suggested that CXCL8 is also implicated in disease-related processes including tissue damage, fibronolysis, angiogenesis and tumorigenesis, suggesting that CXCL8 may be implicated in disease pathologies in which inflammation plays a vital role [11].

It is well accepted that inflammation as a predominant regulator is involved in the developmental processes of numerous human cancers, and is the seventh leading of hallmark of cancer [15]. Studies showed that many chronic inflammatory states obviously increase the risk of certain cancers, and about 20% of cancers are thought to be caused by chronic inflammation or inflammatory states [16]. As a pro-inflammatory chemokine, CXCL8 belongs to ELR+ CXC family of chemokines, which shares structural homology with CXCL5. To date, increasing number of studies indicated that CXCL8 and its receptors were overexpressed in several types of human cancers, including colorectal cancer [17], prostate cancer [18], cervical cancer [19] and non-small cell lung cancer [20]. In the current investigation, we found that CXCL8 was significantly up-regulated in tissues with liver cancer, and its overexpression was closely correlated with high clinical stage and tumor infiltration. In agreement with this, it was demonstrated that the expression of CXCL8 was significantly increased in tissues with oesophageal squamous cell carcinoma, of which stronger expression of IL-8 predominantly connected with many advanced-stage pathological characteristics, including depth of invasion, lymph node metastasis, pathologic stage, lymphatic invasion and venous invasion [21]. Recently, CXCL8 was also proposed to be predominately overexpressed in tissues with bladder cancer, its overexpression was tightly associated with advanced disease, and the overall survival rate of patients with increased expression of CXCL8 was obviously reduced [22]. These findings suggest a possible significance of CXCL8 in cancer development and progression.

Accumulating studies have revealed that CXCL8 is a critical component that involved in tumor initiation, promotion and progression. In cervical cancer, Jia et al. reported that the proliferation and migration of HeLa cervical cancer cells were significantly enhanced after cells treated with different concentrations of exogenous CXCL8 [19]. Accordingly, CXCL8 is also responsible for cancer cells malignant behavior in an autocrine fashion. For example, PC-3 prostate cancer cells overexpressing CXCL8 present rapidly tumorigenicity, highly proliferation rate, remarkably angiogenesis, and exhibit 100% incidence of lymph node metastasis [23]. This investigation also demonstrated that several genes associated with angiogenesis and metastasis, including VGGF, MMP-2 and MMP-9, were up-regulated in the clones with high CXCL8 expression. On the contrary, one study suggested that neutralizing antibodies against CXCL8 exerted a partial inhibition of tumor growth and exhibited anti-angiogenesis activity in a nude mouse xenografts model [24].

However, it is not clear whether CXCL8 stimulates the malignant process of liver cancer. In the present study, we demonstrated that exogenous administration of CXCL8 significantly promote HepG2 cells proliferation and migration, and overexpression of CXCL8 can also facilitate to these behaviors through an autocrine pathway. Finally, mechanism studies demonstrated that overexpression of CXCL8 in HepG2 cells regulates tumor-specific protein expression including ERK1/2, BAX and survivin. The involvement of CXCL8 in tumor progression and angiogenesis were mediated by multifaceted signaling pathways including NF-κB, JNK, PI3K/Akt, p38 MAPK and ERK [25–27], in which ERK signal pathway is a crucial mediator of a variety of cancer cells fates including proliferation, migration and survival. In lung cancer, it was suggested that EGF stimulated a significant increase of CXCL8 production in lung cancer cells, and IL-8 production from lung cancer cells could be initiated by their own produced factors, resulting in the recruitment of inflammatory cells in the tumor microenvironment, as well as the formation of inflammatory microenvironment through PI3K/Akt and ERK pathways [28]. CXCL8 signaling has also been proposed to have a vital role in promoting tumor progression, by regulating apoptosis-related gene expression. For example, administration of the anti-cancer reagent induced CXCL8 production, and the expression of the receptors of CXCL8, CXCR1 and CXCR2. In addition, CXCL8-mediated chemoresistance to oxaliplatin was demonstrated to be mediated by induction of NFκB-transcription, leading to the up-regulation of various anti-apoptotic genes, including Bcl-2 and survivin [29].

## Conclusions

We showed that CXCL8 expression was up-regulated in tissues with liver cancer, exogenous administration and overexpression of CXCL8 significantly facilitated to the malignant phenotypes of HepG2 cells by regulating tumor-specific protein expression including ERK1/2, survivin, caspase-3 and BAX. Our findings might provide a potential marker and target for the treatment and diagnosis of liver cancer.

## Data Availability

The supplementary information that accompanies this article can be accessed via the corresponding author.

## Abbreviations

HBV: hepatitis B virus
HCV: hepatitis C virus
OD: optical density
